# Effects of glucosamine-chondroitin combination on synovial fluid IL-1β, IL-6, TNF-α and PGE2 levels in internal derangements of temporomandibular joint

**DOI:** 10.4317/medoral.20242

**Published:** 2015-02-07

**Authors:** İbrahim Damlar, Emin Esen, Ufuk Tatli

**Affiliations:** 1 DDS, PhD. Assistant Professor; Department of Oral and Maxillofacial Surgery Faculty of Dentistry, Mustafa Kemal University, Hatay, Turkey; 2 DDS, PhD. Professor of Oral and Maxillofacial Surgery; Private Practice, Adana, Turkey; 3 DDS, PhD, Assistant Professor; Department of Oral and Maxillofacial Surgery Faculty of Dentistry, Çukurova University, Adana Turkey

## Abstract

**Background:**

The aim of the present study was to evaluate the effects of glucosamine-chondroitin sulphate combination on internal derangements of temporomandibular joint in clinical and biochemical manners.

**Material and Methods:**

This randomized clinical study included 31 cases reporting joint tenderness, in which disc displacement was detected on MR imaging. In all patients, synovial fluid sampling was performed under local anesthesia. In the study group, the patients were prescribed a combination of 1500 mg glucosamine and 1200 mg chondroitin sulphate, while patients in the control group were only prescribed 50 mg tramadol HCl (twice daily) for pain control. After 8 weeks, synovial fluid sampling was repeated in the same manner. The levels of pain, maximum mouth opening (MMO), synovial fluid IL-1ß, IL-6, TNF-α and PGE2 measured before and after pharmacological intervention were compared.

**Results:**

The reduction in pain levels was significant in both groups. There was no significant difference between two groups in terms of pain reduction. The improvement in MMO was significant in the study group but it was not in the control group. The MMO improvement was significantly higher in the study group compared to the control group. In the study group, significant decrease was observed in PGE2 level, while the decreases in IL-1β, IL-6 and TNF-α levels were not significant. In the control group, no significant decrease was observed in any of the inflammatory cytokines after 8 weeks, moreover IL-1ß and IL-6 levels were increased. Alterations of IL-1ß and IL-6 levels were significant in study group while TNF-α and PGE2 levels were not, compared to control group.

**Conclusions:**

In conclusion, these results might suggest that glucosamine-chondroitin combination significantly increases the MMO and decreases the synovial fluid IL1β and IL6 levels in internal derangements of TMJ compared to tramadol. The modifications of synovial fluid TNF-α and PGE2 levels do not reach statistical significance. This combination also provides efficient pain relief in similar level with tramadol, a narcotic analgesic.

**Key words:** Chondroitin sulphate, glucosamine, internal derangement, TMJ, tramadol.

## Introduction

Temporomandibular joint (TMJ) disorders become increasingly common in society over years. It has been seen that these disorders result from various etiologies (1). Degeneration of articular structures causes evolution of non-inflammatory disorders to inflammatory ones. These degenerative states enhance tissue destruction and joint dysfunction by causing release of inflammatory mediators to joint space (2).

Increased interleukin 1 beta (IL-1ß), interleukin 6 (IL-6), tumor necrosis factor-alpha (TNF-α) and prostaglandin E2 (PGE2) levels in synovial fluid are seen in internal derangements of TMJ such as anterior disc displacement and osteoarthritis (3-5). In many studies, it has been reported that there was an increase in the levels of these mediators in inflammatory joint diseases (6-8). Also, degeneration in the collagen configuration of joint tissues and reduction in the amount of proteoglycans are seen in these disorders (2).

Glucosamine and chondroitin are structural molecules of joint cartilage (9). Chondroitin is a glycosaminoglycan comprising a part of proteoglycan structure of cartilage. Glucosamine is an essential glycoprotein that is necessary for proteoglycan and glycosaminoglycan synthesis (10). There are only two pilot studies concerning use of glucosamine and chondroitin in TMJ disorders (11,12).

Tramadol is a narcotic analgesic with central activity without any anti-inflammatory effect. It is a synthetic agent belonging to weak opiates class which has both opiate and non-opiate mechanisms of action (13). It has been recommended in the management of pain related to inflammatory joint diseases (e.g. osteoarthritis) (14,15). However, to the best of our knowledge, there is no study on the use of tramadol HCl in the complaints related to TMJ.

The aim of the present randomized clinical study was to evaluate the effects of glucosamine chondroitin sulphate combination on the internal derangements of temporomandibular joint and also to compare the effects of this combination with tramadol, a narcotic analgesic, in clinical and biochemical manners.

## Material and Method

**Figure 1:**
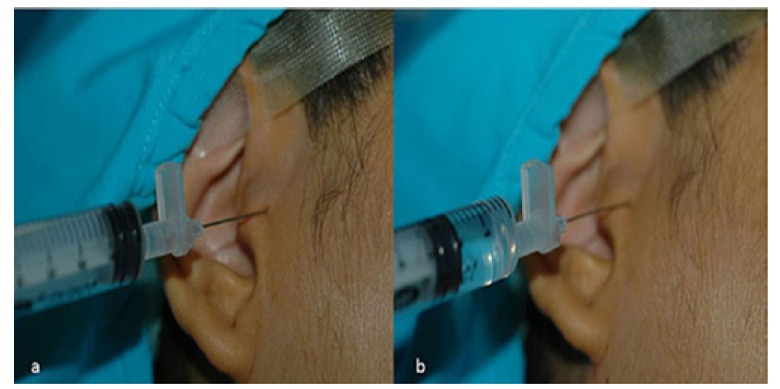
Injection (a) and aspiration (b) of saline into upper joint space of TMJ.

The study was approved by Institutional Ethics Committee. All participants were informed in detail and signed a consent form. The study was conducted on 34 women (35 joints) aged 18-40 years (mean age 28.6 years±6.89) with internal derangement of TMJ including Wilkes II or III (16), pain (longer than 4 weeks), and limitation in mouth opening who had anterior disc displacement. The patients were included after clinical and MRI examinations. The patients who were previously treated (any invasive treatment or non-steroidal anti-inflammatory drugs) for TMJ complaints were excluded. Pain levels of the patients during palpation of TMJ were recorded according to a numeric pain scale (0: no pain; 10: intractable pain) (17). The maximum mouth opening (MMO) was measured between the edges of the upper and lower central incisors by a millimetric ruler. Conscious sedation was performed using 0.1 mg/kg i.v. midazolam. Auriculotemporal nerve block was performed by a 35-gauge needle. The needle was inserted at a point just below the earlobe and advanced in an anteriosuperior direction, aiming the posterior aspect of the mandibular condyle; then 2 ml of 5% bupivacaine hydrochloride (Marcaine, AstraZeneca, Turkey) was injected. A 21-gauge needle was inserted to upper joint space via superior posterolateral approach (18). In this technique first needle puncture is 10 mm anterior to the tragus and 2 mm inferior to an imaginary line connecting the tragus and the outer corner of the eye. Then, 2 ml of saline was injected into the joint space, which was aspirated thereafter (Fig. 1). This procedure was repeated for ten times to obtain synovial fluid sample as described previously by Wake *et al*. (19) Samples obtained were stored at -70°C. The patients were randomly assigned into 2 groups. The study group was given a combination of 1500 mg glucosamine and 1200 mg chondroitin sulphate per day (Lifetime®, Nutritional Specialties, Inc. California, USA). The control group was only given 50 mg tramadol HCl twice daily per oral (Contramal, Abdi İbrahim, Turkey) for pain relief. The medications were given to the patients by one of the researcher and the medications were not in their original package. The names of the medications were not explained to the patients formally. The sampling procedures were performed by another researcher who did not know the groups of the patients formally. Moreover; during second sampling procedures, the results of the first samplings were masked. After 8 weeks, synovial fluid samples were obtained in the same manner. In case of blood aspiration during synovial fluid sampling, the patient was excluded. The IL-1ß, IL-6, TNF-α and PGE2 levels were measured in samples by using Enzyme-Linked ImmunoSorbent Assay (ELISA) kit (DIA source Immuno Assays S.A. Rue de l’Industrie, Nivelles-Belgium). The levels of inflammatory cytokines, pain, and MMO between two groups were compared.

All statistical analyses were performed by SPSS for Windows version 18.0 software. Mann Whitney U test was used to compare measurements without normal distribution between groups. Dependent measurements such as before-after were compared by paired samples t-test and Wilcoxon signed rank tests where appropriate. *p*<0.05 was considered as statistically significant in all tests.

## Results

Three subjects were excluded due to blood aspiration and 1 due to vertigo owing to tramadol HCL. No other adverse effect was observed in either of the groups. Inflammatory cytokines were detected in all patients included. It was observed that IL-1ß level was not significantly decreased in the study group (*p*=0.070), whereas it was increased in the control group (*p*=0.004). IL-6 level was decreased in the study group, but the difference did not reach to a statistical significance (*p*=0.063). IL-6 level was increased in the control group, however the corresponding increase was not statistically significant (*p*=0.104). TNF-α levels were decreased in both groups, which did not reach statistical significance (*p*=0.167 for the study group and *p*=0.840 for the control group). PGE2 level was significantly decreased in the study group (*p*=0.000) but not in the control group (*p*=0.252). It was observed that both agents had significant effects on pain reduction (*p*=0.001 for study group and *p*=0.000 for control group) ( Table 1). In terms of pain relief, the difference between two groups was not statistically significant (*p*=0.770) ( Table 2). It was observed that glucosamine-chondroitin combination and tramadol had significant effect on MMO improvement (*p*= 0.024 for study group, and *p*= 0.00 for control group) ( Table 1). The improvement of MMO was significantly higher in glucosamine-chondroitin combination group when compared with tramadol group (*p*=0.000) ( Table 2).

**Table 1:**
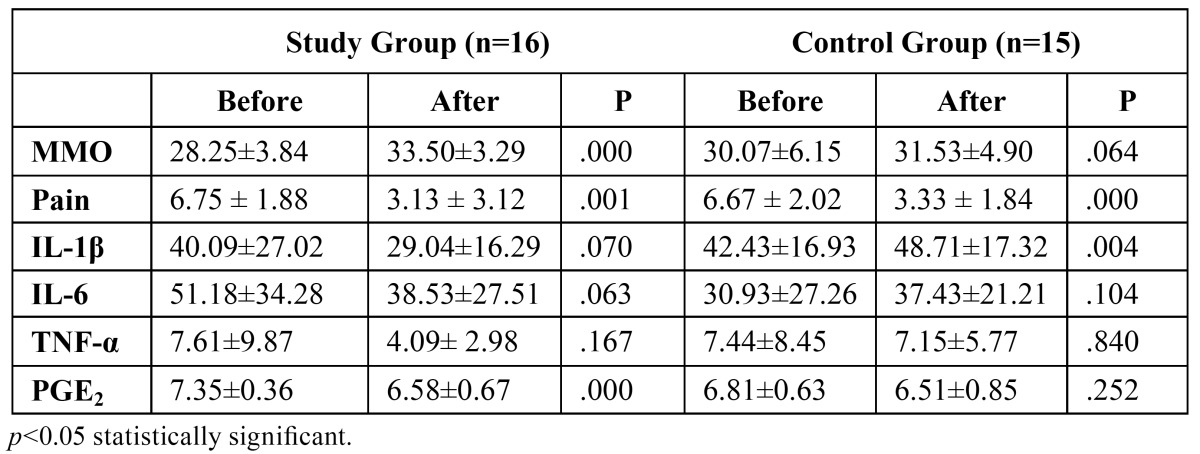
Maximum mouth opening (MMO) (mm), pain (numeric scale) and cytokine levels (pg/mL) before and after experiment in both groups.

**Table 2:**
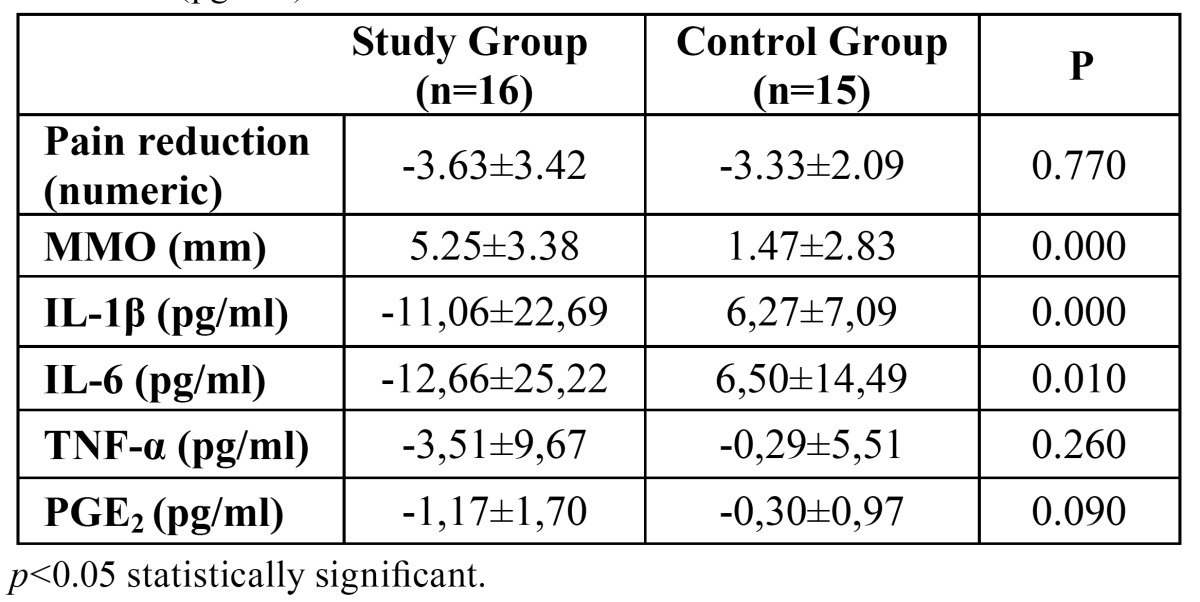
Comparison of two groups in terms of improvement of maximum mouth opening (mm), pain reduction (numeric scale) and cytokine levels difference (pg/mL).

## Discussion

In this randomized, double-blinded, controlled clinical study, we evaluated the effects of glucosamine-chondroitin sulphate combination on synovial fluid IL-1ß, IL-6, TNF-α and PGE2 levels in internal derangement of TMJ.

The inflammatory cytokines such as prostaglandins, interleukines and TNF-α are potential regulators of osteoclastogenesis (20). Increased synovial fluid IL-1ß, IL-6 and TNF-α levels, which are important findings of inflammation, have been frequently addressed in the literature (2,20-22). Kaneyama *et al*. (22) compared IL-1ß, IL-6 and TNF-α levels measured in synovial fluid of TMJ in 55 patients with osteoarthritis and 5 healthy volunteers. Authors found that levels of inflammatory mediators were higher in patients with internal derangement compared to healthy individuals. In another study by the same authors (21), synovial fluids obtained from 57 patients with degenerative joint disease were compared to those obtained from 7 healthy individuals. Authors concluded that the inflammatory mediators were associated with TMJ abnormalities. In particular, they reported that IL-6 plays a major role in the development of osteoarthritis. The values of cytokines in the present study differ widely from the above mentioned two studies. It is difficult to clarify the reason while we have great hassle to standardize the sampling methods, assay methods, and patients (22). The results might be affected by various variables including patients’ age and gender distribution, duration and intensity of the TMJ diseases, trademark of used ELISA kits etc.

Quinn and Bazan (23) were the first to determine the presence of PGE2 in inflamed synovial fluid of TMJ and showed a considerable correlation with levels of acute synovitis. In our study, these mediators were detected in all symptomatic patients with internal derangement of TMJ detected on MR imaging. The decrease in the levels of these mediators in the synovial fluids of the study group indicates the anti-inflammatory effect of glucosamine-chondroitin sulphate.

There are many studies suggesting the use of glucosamine and chondroitin in the management of osteoarthritis in the literature (24-26). The majority of these studies address its use in knee and hip osteoarthritis; however, there are only two pilot studies concerning TMJ disorders (11,12). In 1988, Shankland *et al*. (11) reported that these molecules decreased the sound and pain in TMJs. In that study, a decrease was observed in the sound and pain of joints in patients taking glucosamine and chondroitin sulphate but authors suggested that a double-blinded study with NSAI control would provide more significant results. In 2001, Nguyen *et al*. (12) reported that glucosamine and chondroitin decreased TMJ pain and consumption of other analgesics by the patients. In our study, a decrease was detected in pain level and synovial fluid IL-1ß, IL-6, TNF-α and PGE2 levels in patients using 1500 glucosamine and 1200 mg chondroitin. Moreover, a significant improvement in MMO was also observed in patients using glucosamine-chondroitin combination. Given that inflammatory mediators appear to be related to cartilage destruction, it might be suggested that glucosamine and chondroitin diminishes cartilage degeneration while efficiently reducing pain in similar level with tramadol, a narcotic analgesic with central activity.

Long-term use of non-steroid anti-inflammatory (NSAI) drugs in painful internal derangements of TMJ may result in severe gastrointestinal complications. Simanek *et al*. (27) noted that glucosamine and chondroitin should be considered as a nutritional supplement rather than a drug, since they have fewer side effects which can be neglected. In a study in which effects of glucosamine and chondroitin sulphate on bovine cartilage culture were evaluated in vitro, Chan *et al*. (28) showed that glucosamine-chondroitin combination decreased matrix metalloproteinase and nitric oxide production. In the same experiment, it was observed that these molecules inhibited mRNA translocation which would inhibit inflammatory cytokines and collagenase production.

Tramadol HCl is a synthetic analgesic with central activity, without anti-inflammatory effect, which is used in cancer, back pain, neuropathic pain and pain related to musculoskeletal disorders such as osteoarthritis (13,29). American Pain Society (APS) recommends tramadol HCl alone or in combination with acetaminophen or NSAI drugs to relieve pain related to osteoarthritis (14). In the present study, tramadol HCl was used in the control group for pain relief. Pubmed search reveals no study evaluating the effects of tramadol HCl in TMJ disorders. In a multi-center, randomized, double-blinded study on 1028 patients, Burch *et al*. (13) compared the effects of tramadol HCl with those of placebo in pain related to osteoarthritis. It was reported that a dose of 200 mg tramadol HCl per day caused significant reduction in pain when compared to placebo. In that double-blinded study; nausea was observed in 66%, followed by constipation in 61%, vertigo in 42%, and sleepiness in 29% of the patients. These rates were in accordance with the literature in 3 months therapy (30). Tramadol has been widely prescribed for over 4 decades in many countries with limited evidence of dependence and abuse. It may be effective in alleviating spontaneous opioid withdrawal, although doses higher than those typically prescribed for analgesia may be required (31). In our study, one patient was excluded who failed to use tramadol due to vertigo. In patients using 100 mg tramadol HCl, an insignificant increase in IL-1ß and IL-6 levels, and an insignificant decrease in TNF-α and PGE2 levels were observed, which confirmed tramadol’s central analgesic utility with no anti-inflammatory effect.

In conclusion, these results might suggest that glucosamine-chondroitin combination significantly increases the MMO and decreases the synovial fluid IL1β and IL6 levels in internal derangements of TMJ compared to tramadol. In the glucosa-mine-chondroitin combination group, the modifications of synovial fluid TNF-α and PGE2 levels do not reach statistical significance compared to tramadol group. This combination also provides efficient pain relief in similar level with tramadol, a narcotic analgesic. As a nutritional supplement with little side effects compared to NSAI or narcotics, glucosamine-chondroitin combination might be considered as an adjunctive agent in the treatment of internal derangements of TMJ.
